# Interspecific hybridisation provides a low-risk option for increasing genetic diversity of reef-building corals

**DOI:** 10.1242/bio.060482

**Published:** 2024-08-29

**Authors:** Annika M. Lamb, Lesa M. Peplow, Ashley M. Dungan, Sophie N. Ferguson, Peter L. Harrison, Craig A. Humphrey, Guy A. McCutchan, Matthew R. Nitschke, Madeleine J. H. van Oppen

**Affiliations:** ^1^ Australian Institute of Marine Science, 1526 Cape Cleveland Road, Cape Cleveland 4810, Queensland, Australia; ^2^ School of Biosciences, The University of Melbourne, Grattan Street, Parkville VIC 3010; ^3^ AIMS@JCU - James Cook University, Townsville, QLD 4811, Australia; ^4^ Marine Ecology Research Centre - Southern Cross University, Lismore, NSW 2480

**Keywords:** *Acropora*, Coral reef, Great Barrier Reef, Symbiodiniaceae, Hybrid

## Abstract

Interspecific hybridisation increases genetic diversity and has played a significant role in the evolution of corals in the genus *Acropora*. *In vitro* fertilisation can be used to increase the frequency of hybridisation among corals, potentially enhancing their ability to adapt to climate change. Here, we assessed the field performance of hybrids derived from the highly cross-fertile coral species *Acropora sarmentosa* and *Acropora florida* from the Great Barrier Reef. Following outplanting to an inshore reef environment, the 10-month survivorship of the hybrid offspring groups was intermediate between that of the purebred groups, although not all pairwise comparisons were statistically significant. The *A. florida* purebreds, which had the lowest survivorship, were significantly larger at 10 months post-deployment compared to the other three groups. The four offspring groups harboured the same intracellular photosymbiont communities (Symbiodiniaceae), indicating that observed performance differences were due to the coral host and not photosymbiont communities. The limited differences in the performance of the groups and the lack of outbreeding depression of the F1 hybrids in the field suggest that interspecific hybridisation may be a useful method to boost the genetic diversity, and as such increase the adaptive capacity, of coral stock for restoration of degraded and potentially genetically eroded populations.

## Teaser

**Summary:** Viability of coral hybrids in the ocean indicates that they could be a useful tool for reef conservation.

## INTRODUCTION

Coral reef degradation can be attributed to a range of human disturbances including climate change (Doney et al., 2009; De'ath et al., 2012; Souter et al., 2021). Stressful conditions cause reef-building corals to lose their microalgal symbionts (Symbiodiniaceae) through a process termed coral bleaching and prolonged or severe stress often results in them dying (Hoegh-Guldberg, 1999). Drastic and rapid declines in coral cover have been observed on many reefs and suggest that the existing resilience of corals, their ability to change their behaviour, morphology, and physiology (phenotypic plasticity), and their rate of adaptation may be insufficient to ensure their persistence into the future (Pandolfi et al., 2003; van Hooidonk et al., 2016). Thus, human interventions may be required to bridge the gap while global action is taken to minimise further climate change (Rinkevich, 2005; de Groot et al., 2012).

Reef restoration by outplanting coral stock is one intervention to supplement populations and counter reef degradation (Boström-Einarsson et al., 2020; Knowlton et al., 2021). Improvements in our understanding of the sexual breeding cycle of corals increase the feasibility of generating large numbers of corals for restoration programs (Randall et al., 2020; Banaszak et al., 2023). Managed breeding involves interbreeding corals in a controlled way to maximise genetic diversity and/or tolerance of coral stock and can be incorporated into restoration programs to maximise the resilience and adaptive capacity of recipient reefs (National Academies of Sciences, 2019).

One way in which the diversity and tolerance of corals might be maximised through managed breeding is by combining the gametes of compatible pairs of species to conduct interspecific hybridisation (Chan et al., 2019a). Interspecific hybridisation generates unique genetic combinations that can confer resilience to various conditions and breaks genetic correlations that constrain evolution (Carlson et al., 2014). As a demonstration of this, the expansion of hybrids into novel niches has been implicated in the diversification and adaptive radiation of African cichlid fish (Meier et al., 2017), Darwin's finches (Lamichhaney et al., 2015), fruit flies (Feder et al., 2005), and Hawaiian silverswords (Barrier et al., 1999, see Meier et al., 2017 for mechanisms). Interspecific hybridisation can reduce the risk of extinction by increasing the genetic diversity and thus adaptive potential of species (Chan et al., 2019a).

Coral species share genetic signatures among their genomes that indicate they have semi-permeable boundaries and naturally interbreed (Mao et al., 2018; van Oppen et al., 2002). In the Caribbean, *Acropora palmata* and *Acropora cervicornis* interbreed to produce the F1 hybrid *Acropora prolifera* (van Oppen et al., 2000; Nylander-Asplin et al., 2021). Putative fixed genetic differences have been identified in functional genes of *A. palmata* and *A. cervicornis*, which are crossed to generate unique genetic combinations in the *A. prolifera* hybrids (Kitchen et al., 2019; Kitchen et al., 2020). The unique genetic combinations in the *A. prolifera* genome may explain its capacity to exist outside the range of its purebred parental species (Fogarty, 2012).

Interspecific hybridisation between coral species has been successfully conducted in the laboratory through *in vitro* fertilisation (Hatta et al., 1999; Willis et al., 1997; van Oppen et al., 2002). Chan et al. (2018) conducted *in vitro* hybridisation in pairs of acroporids and further demonstrated that *Acropora* hybrids can be equally or more resilient to elevated temperatures and *p*CO_2_ levels than their purebred counterparts in a laboratory environment. These lines of evidence support the notion that interspecific hybridisation can produce resilient coral stock that may be considered for use in coral reef restoration initiatives. However, there are significant outstanding questions that need to be answered to fully understand the potential of interspecific hybridisation as an intervention strategy.

Natural coral reef environments have many environmental fluctuations, predators, symbionts, food sources, and other important ecological variables, as well as interactions between these variables, which cannot be simulated in the laboratory. As such, aquarium experiments impose selective pressures that do not occur in nature. Ecological trade-offs can further result in corals performing well in aquaria but poorly on reefs, or vice versa. Furthermore, there are concerns that first or later generation interspecific hybrids may have reduced fitness relative to both of their purebred counterparts in one or more environments due to incompatibilities between the genomes of the parental species, referred to as outbreeding depression (Frankham et al., 2011). Alternatively, if interspecific hybrids display overdominance, where their fitness is greater than that of both of their parental purebred species, then there is the risk they become invasive throughout natural systems (Ellstrand and Schierenbeck, 2000). If hybrid corals are to be actively used to restore coral populations, then their performance in the ocean must first be evaluated.

Here we compare the survivorship, size, and colour (as a proxy for bleaching status) of interspecific hybrids of *A. florida* and *A. sarmentosa* and their purebred counterparts in the field. We hypothesised that *A. florida* and *A. sarmentosa* interspecific hybrids would perform equally well as or better than their purebred counterparts in the ocean, since this trend has been observed for these species in the laboratory (Chan et al., 2018). This study provides important information for the assessment of the value of hybridisation as a tool to enhance coral adaptive capacity and climate resilience in reef restoration initiatives.

## RESULTS

### Spawning behaviour, fertilisation success, and survivorship in captivity

**Fig. 1.**
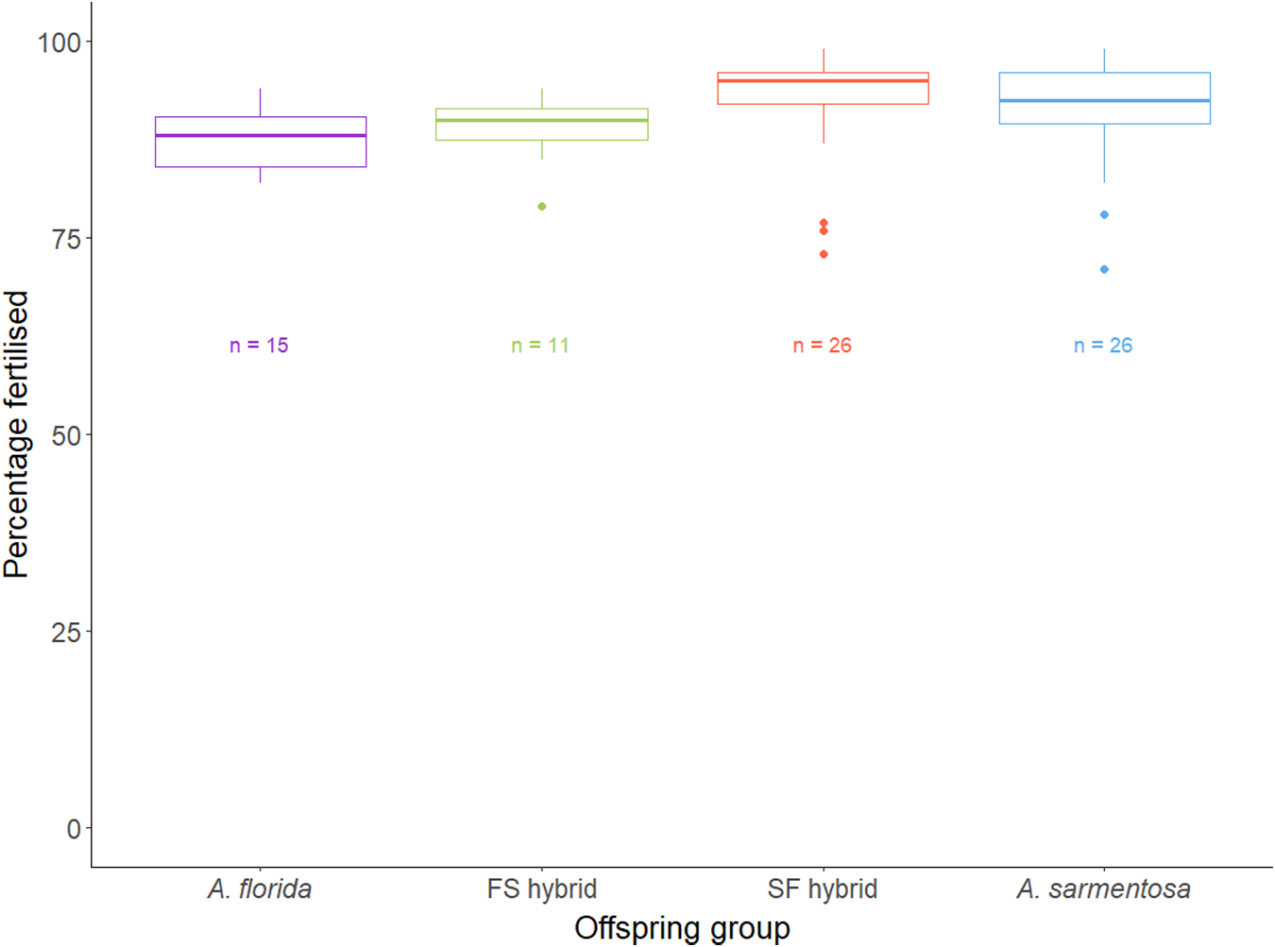
Box plots depicting the distribution of fertilisation success (percentage of multicell embryos at 2.5 h post fertilisation) of each of the offspring groups. The horizontal lines of the boxes represent the lower quartile, median, and upper quartile values, the whiskers represent the extreme values and dots represent single outlier datapoints. Sample sizes (number of fertilisation counts) are shown below each box for each offspring group.

Five *A. florida* and nine *A. sarmentosa* colonies collected from Davies Reef began spawning between 20:15–20:30 h on the 20/11/2019 and were crossed following Chan et al. (2018). The eggs and sperm of the parental colonies were combined to generate two hybrid offspring groups: *A. florida* eggs were crossed with *A. sarmentosa* sperm to produce FS hybrids and *A. sarmentosa* eggs were crossed with *A. florida* sperm to produce SF hybrids (Table 1). The eggs and sperm of conspecific colonies were crossed to produce *A. florida* and *A. sarmentosa* purebred controls (Table 1). There was no significant difference in fertilisation success among the offspring groups (z-values −0.530–1.195; *P* values 0.623–0.994; Fig. 1). Early life (until deployment at 10 months post settlement) survivorship of the *A. florida*, FS hybrids, SF hybrids, and *A. sarmentosa* recruits in captivity varied between 1.7–27.7%, 2.4–59.7%, 3.7–21.2%, and 14.2–33.5%, respectively, among three rearing tanks. There was no significant difference in early life survivorship amongst the offspring groups (z-ratio=−1.66–2.25, *P>*0.149).

**Table 1.**
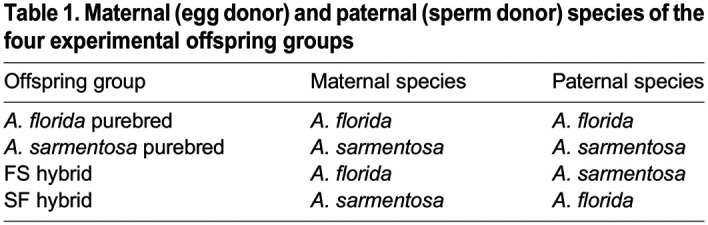
Maternal (egg donor) and paternal (sperm donor) species of the four experimental offspring groups

### Post-deployment performance

The 70 *A. florida* purebred, 110 FS hybrid, 40 SF hybrid, and 93 *A. sarmentosa* purebred corals growing on tiles were distributed among four frames in Geoffrey Bay, Yunbenun, when they were 10 months of age (Fig. S1). Upon deployment, the densities of the corals on the tiles were similar amongst the offspring groups: the mean number of corals per deployed tile was 2.4, 2.4, 1.7, and 2.3 for the *A. florida* purebred, FS hybrid, SF hybrids, and *A. sarmentosa* purebred corals, respectively. The performance of the hybrid and purebred offspring groups was assessed prior to deployment and then approximately two (November 2020), four (January 2021), six (March 2021), and 10 months (July 2021) post-deployment. The survivorship, size, and colour of the four offspring groups were compared over time.

#### Survivorship

Bayesian generalised linear mixed effects models (BGLMMs) were built to test the effect of time, offspring group, and an interaction between the two on coral survivorship in the field. The results of the best performing BGLMM (see Table S1 for model comparisons) demonstrated that *A. florida* purebreds had lower survivorship than the *A. sarmentosa* purebreds at two months [difference in estimate marginal means (ΔEMM)=−3.30, 95% highest posterior density interval (HPD)=−6.27 – −0.3]) and six months (ΔEMM=−3.616, HPD=−6.90 – −0.09) post-deployment; all other pairwise comparisons of survivorship amongst offspring groups at each time point were not significant. The R-hat convergence diagnostics of the best performing BGLMM were <1.05, the bulk effective samples sizes (1803–2511) and tail effective sample sizes (1859–2416) of the model estimates were large, the posterior distributions of the model estimates were unimodal and normally-distributed, and the time-series plots of the model estimates for each chain tracked with one another, indicating that the model fit the data and that the chains converged. A model that accounted for the repeated measures design and included the fixed effects of time (continuous), offspring group, and an interaction between the two fit the data better than models that also included the random effects of tile and/or frame (Table S1).

**Fig. 2.**
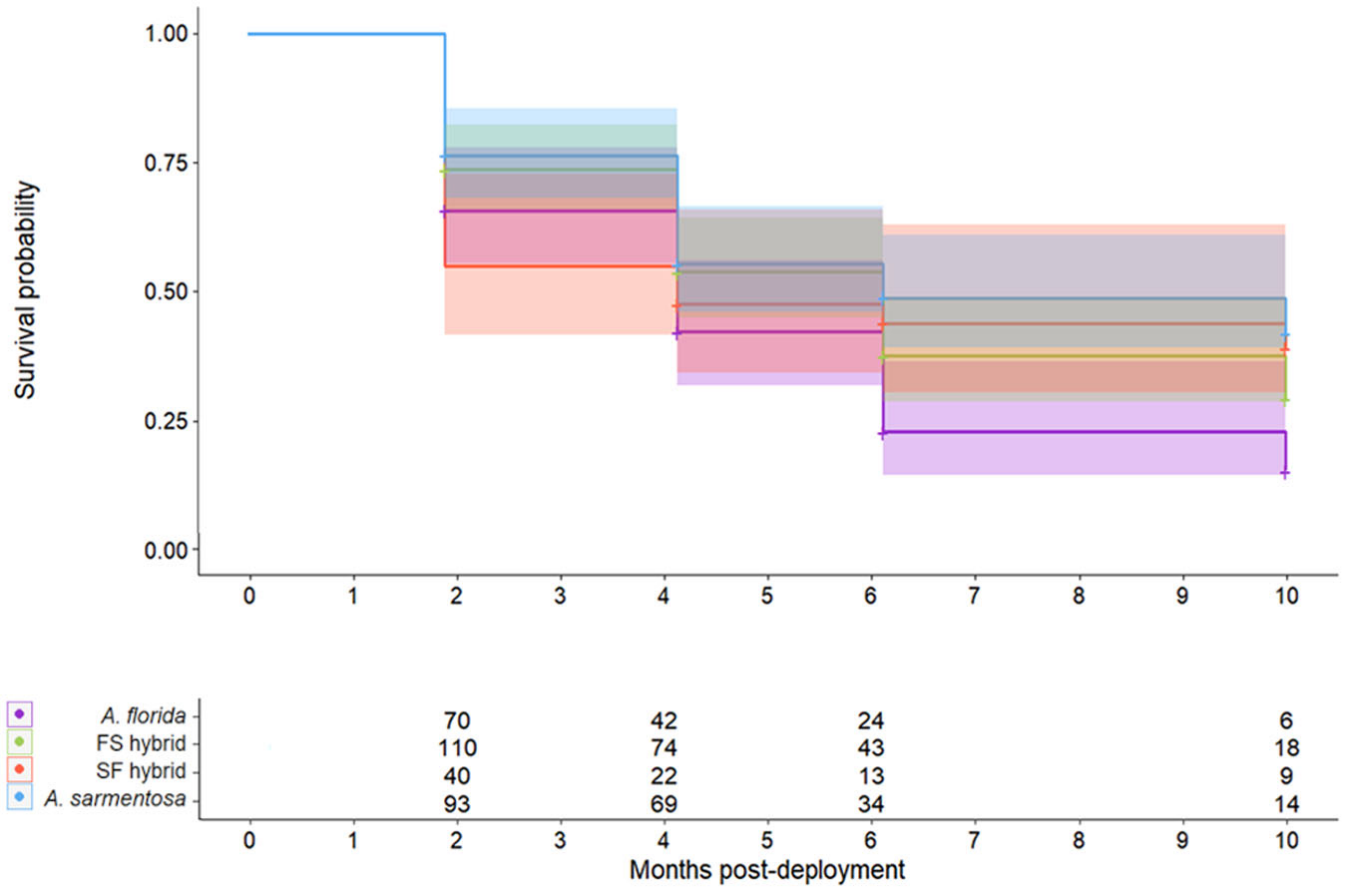
**The probability of recruits surviving over the 10-month reef deployment shown for each of the offspring groups.** (*A. florida* purebred – purple, FS hybrid – green, SF hybrid – red, and *A. sarmentosa* purebred – blue). The data points on the graph represent the mean probability and the upper and lower vertical limits of the shaded areas represent the 95% confidence intervals around the mean. Sample sizes (number of recruits that were counted as dead or alive) are shown in the table for each offspring group and timepoint.

The BGLMM comparisons indicated that the random variance among frames and tiles was insignificant relative to the variation among individuals (Table S1). Therefore, Kaplan–Meier survival probabilities (which do not account for random effects) were considered valid statistical measures to compare the survivorship among the offspring groups. The following results relate to the log-rank test comparisons among offspring groups of the Kaplan–Meier estimates of survival probability. A significant difference in survivorship among offspring groups was detected (*P*=0.041; Fig. 2). *Acropora sarmentosa* purebreds had better survivorship throughout the deployment than the *A. florida* purebreds (*P*=0.023; Fig. 2). FS hybrids (*P*=0.146) and SF hybrids (*P*=0.373) showed a trend of better survivorship than *A. florida* purebreds, but neither comparison was statistically significant (Fig. 2). No significant difference was detected in the survivorship of FS hybrids and *A. sarmentosa* purebreds (*P*=0.373; Fig. 2), SF hybrids and *A. sarmentosa* purebreds (*P*=0.410; Fig. 2), or FS and SF hybrids (*P*=0.937; Fig. 2). At 10 months post-deployment, the probability of survivorship of the *A. florida* purebreds, FS hybrids, SF hybrids, and *A. sarmentosa* purebreds was 15.3% (CI=7.3–31.7%), 29.2% (CI=20.3–41.9%), 39.0% (CI=25.4–59.9%), and 41.8% (CI=30.8–56.9%), respectively.

#### Size

**Fig. 3.**
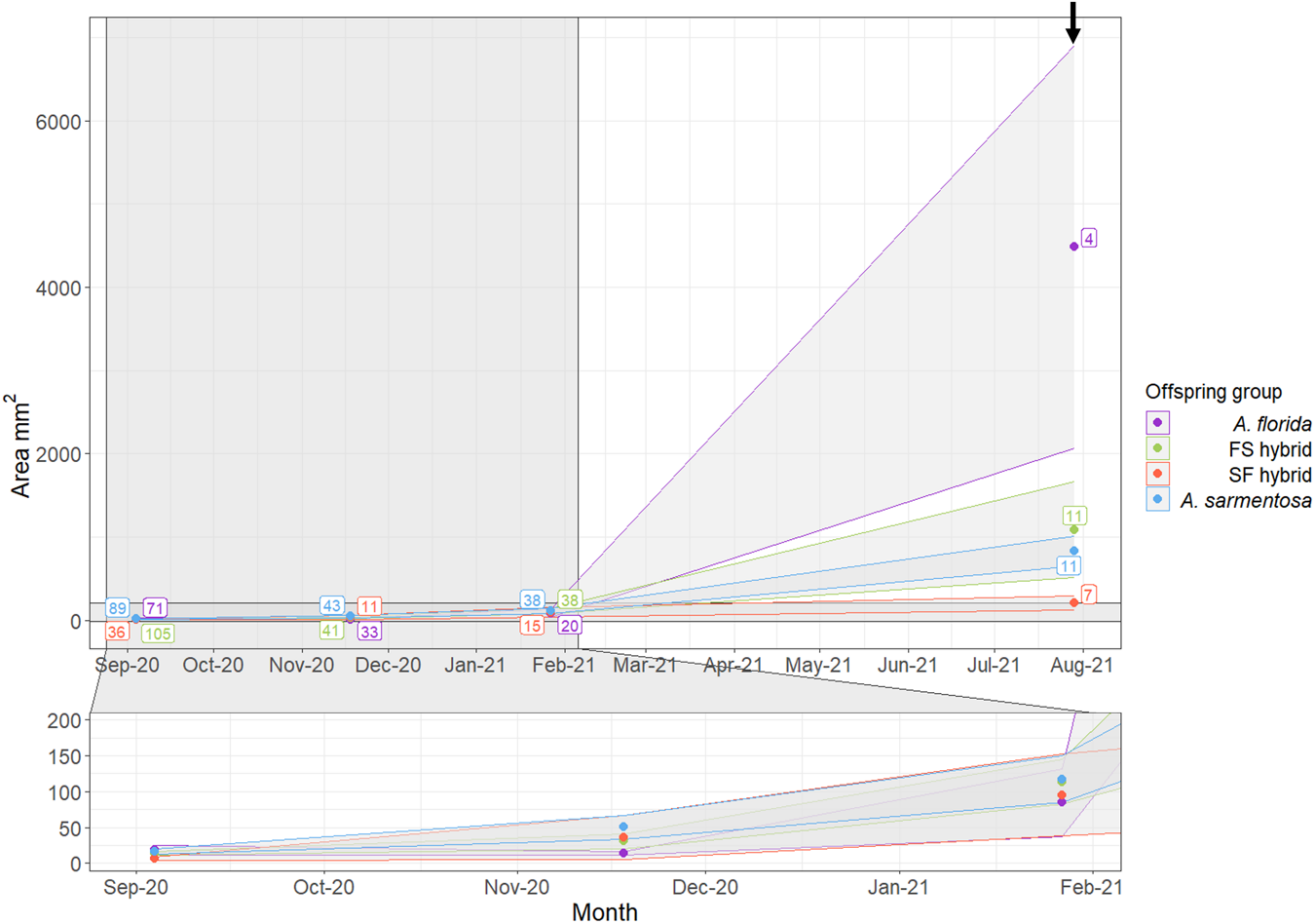
**Recruit surface area (mm^2^) over the 10 months post-deployment shown for each of the offspring groups.** The data points represent the mean area (mm^2^) and the upper and lower limits of each ribbon represent the standard error around the mean. Sample sizes (number of recruits) are included in boxes next to the data points. The 10-month post-deployment size estimates (annotated with an arrow) were obtained through 3D modelling whilst sizes from earlier time points were estimated using 2D imaging.

Linear mixed effects modelling demonstrated that the *A. florida*, FS hybrid, and *A. sarmentosa* recruits grew significantly between 4 and 10 months post-deployment (ΔEMM=726.98–4133.64, t-ratio=4.88–17.33, *P<*0.001), but the SF hybrids did not change in size (ΔEMM=112.13, t-ratio=0.56, *P=*1.000; Fig. 3). No significant difference in size was detected in any offspring group between deployment and two months post-deployment (ΔEMM=29.25–47.83, t-ratio=0.19–0.56, *P=*1.000) or between 2 and 4 months post-deployment (ΔEMM=29.84–61.42, t-ratio=0.31–0.65, *P=*1.000; Fig. 3). No significant difference in size was detected between the offspring groups at any timepoint prior to the 10-month census (ΔEMM=−11.49–70.50, t-ratio=−0.19–0.583, *P*=1.000; Fig. 3). At 10 months post-deployment, there were few surviving recruits modelled for 3D estimations of surface area: the surface areas of four *A. florida*, eleven FS hybrids, seven SF hybrids, and eleven *A. sarmentosa* individuals were measured. The following results pertaining to recruit size at 10 months post-deployment must therefore be interpreted with caution. At 10 months post deployment, *A. florida* recruits were significantly larger than the FS hybrid (ΔEMM=3268.54, t-ratio=12.49, *P*<0.001), SF hybrid (ΔEMM=4091.01, t-ratio=14.50, *P*<0.001), and *A. sarmentosa* (ΔEMM=3465.64, t-ratio=13.26, *P*<0.001) recruits (Fig. 3). The FS hybrids were significantly larger than the SF hybrids at 10 months post-deployment (ΔEMM=822.47, t-ratio=3.73, *P*=0.010; Fig. 3). There was no significant difference in size between the *A. sarmentosa* and FS hybrids (ΔEMM=197.1, t-ratio=1.02, *P*=1.000) or *A. sarmentosa* and SF hybrids (ΔEMM=−625.36, t-ratio=−2.84, *P=*0.185) at 10 months post-deployment (Fig. 3). These results have been drawn from the best-performing linear mixed effects model (LMM) that tested the effect of offspring group and time, and the interaction between the two, on surface area, while accounting for the repeated measures design, the non-independence of corals growing on the same tile and the increase in variance over time. This model (AIC=8468.84) was found to perform as well as a more complex model that also included frame (AIC=8470.86; likelihood ratio=0.02; *P*=0.890) as a random variable, and better than a less complex model that did not account for the random variation amongst tiles (AIC=8486.07; likelihood ratio=19.22; *P*<0.001).

#### Site temperature tracking and bleaching response

**Fig. 4.**
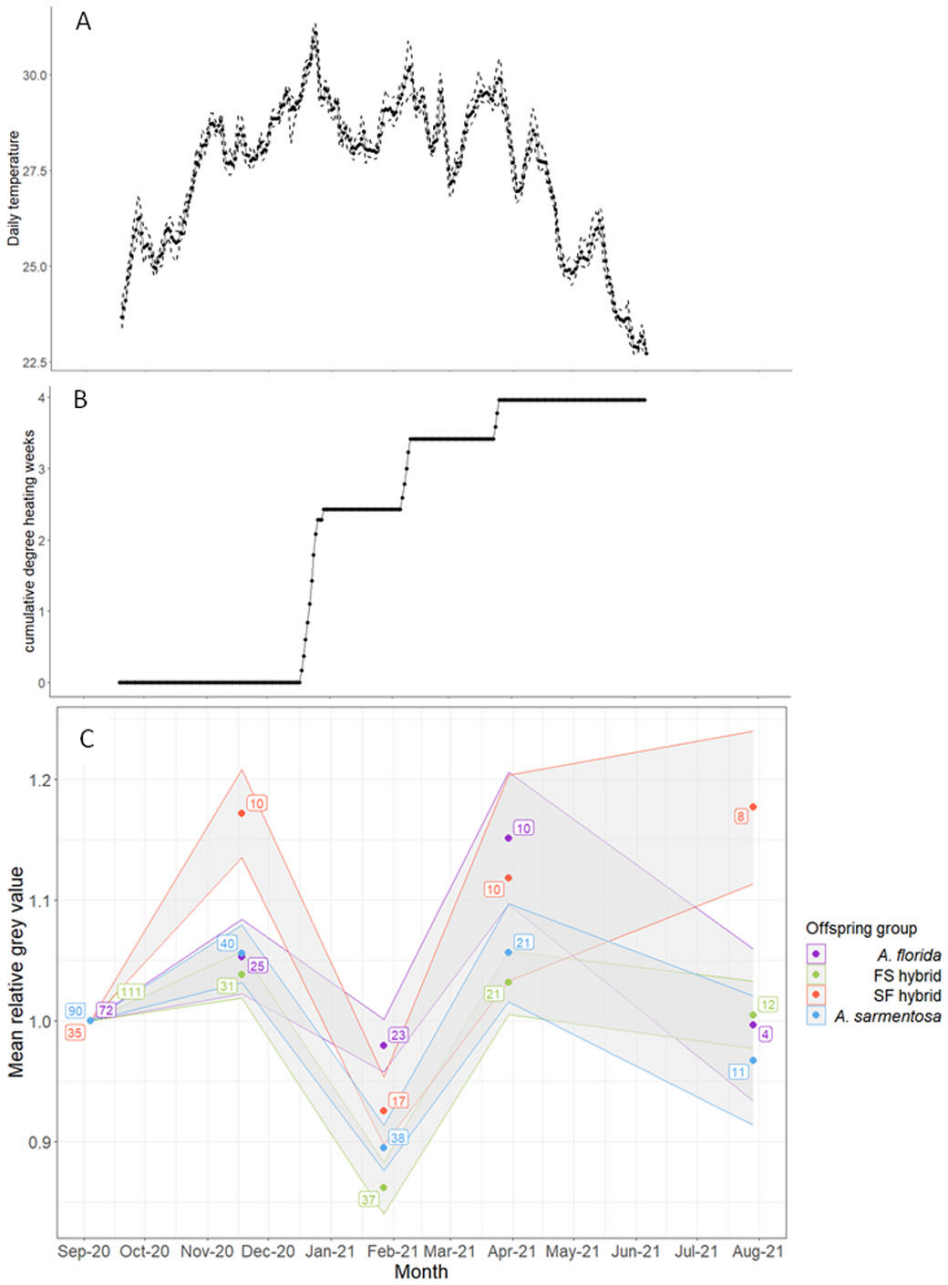
**Observed temperatures at the deployment site.** (A) Mean (solid line), maximum (upper dashed line), and minimum (lower dashed line) daily temperatures (°C) at a 5 m depth in Geoffrey Bay. (B) Cumulative degree heating weeks (°C) relative to the parental colony collection location, Davies Reef, throughout the course of the deployment. (C) Recruit calibrated relative colour scores, derived from grey values (proxy for bleaching) shown for each of the offspring groups over the 10-month deployment from September 2020 to July 2021. The data points represent the colour score relative to the colour score of the recruit at the time of deployment, and the upper and lower limits of each ribbon represent the standard error around the mean. Sample sizes (number of recruits) are included in boxes next to the data points. Colour score is used as a proxy here for the density of algal symbionts in the coral tissue where a lower number/lighter colour can represent a lower algal symbiont density that is indicative of coral bleaching.

The calibrated colour scores, derived from grey values (as a proxy for bleaching) of the corals were tracked over time and examined in relation to the water temperatures and degree heating weeks (DHW) that the corals experienced at the deployment site. DHW is a measurement of the amount of cumulative heat stress experienced by a coral over time based on the historical temperatures experienced by corals at its ancestral reef. Summer temperatures at the inshore Yunbenun reefs are generally warmer than those at the mid-shelf Davies Reef where the parents of the offspring groups tested here were sourced. As a result, relative to Davies Reef, the recruits at Yunbenun experienced three summer heat waves over the course of the experiment in late December 2020, early February 2021, and late March 2021 that were equivalent to 2.43, 0.98, and 0.55 DHWs, respectively, and 3.96 DHWs cumulatively (Fig. 4).

The colouration (relative colour scores) of the recruits were tracked over time and used as a proxy for the density of algal symbionts in the coral tissue where a lower number/lighter colour can indicate a lower algal symbiont density that is indicative of coral bleaching. Linear mixed effects modelling indicated that *A. florida* (ΔEMM=0.05; t-ratio=2.44; *P*=0.062) and FS hybrid recruits (ΔEMM=0.04; t-ratio=2.02; *P*=0.177) showed no significant change in colouration between September (mean daily temperature (MDT)=23.92°C) and November 2020 (MDT=28.41°C), while the SF hybrids (ΔEMM=0.17; t-ratio=4.88; *P*<0.001) and *A. sarmentosa* purebreds (ΔEMM=0.06; t-ratio=3.28; *P*=0.005) darkened (Fig. 4). All offspring groups paled between November 2020 (MDT=28.41°C) and January 2021 (MDT 29.06°C; ΔEMM =-0.24 – −0.07; t-ratio=−7.51 – −2.53; *P*-value<0.001–0.047) and darkened between January (MDT=29.06°C) and March 2021 (MDT=28.52°C; ΔEMM=0.16–0.19; t-ratio=4.87–6.50; *P*<0.001). The FS hybrid, SF hybrid, and *A. sarmentosa* purebred recruits (ΔEMM=−0.09–0.07; t-ratio=−2.46–1.65; *P*=0.058–1.000) showed no difference in colour between March and July 2021 while the *A. florida* recruits (ΔEMM=−0.15; t-ratio=−2.71; *P*=0.029) paled (Fig. 4). The LMM that tested the effect of offspring group and time, and the interaction between the two, on recruit colour, while accounting for the repeated measures design and the non-independence of corals growing on the same tile (AIC=−985.03) fit the data best; this model outperformed a model that did not account for the random variation among tiles (AIC=−965.07; likelihood ratio=21.96; *P*<0.001) and performed as well as a more complex model that also accounted for the variation among frames (AIC=−983.03; likelihood ratio<0.01; *P*=1.00).

#### Summary of post-deployment performance

The FS and SF hybrids had intermediate survivorship compared to the *A. florida* and *A. sarmentosa* purebreds (Table 2). The FS and SF hybrids were smaller than the *A. florida* purebreds and the same size as the *A. sarmentosa* purebreds after the 10-month deployment (Table 2). The two hybrid groups therefore displayed no significant outbreeding depression or overdominance compared to both purebred groups throughout the deployment with respect to their survivorship and size. Finally, all offspring paled at a time that correlated with an extreme heatwave in December 2020 (Table 2).

**Table 2.**
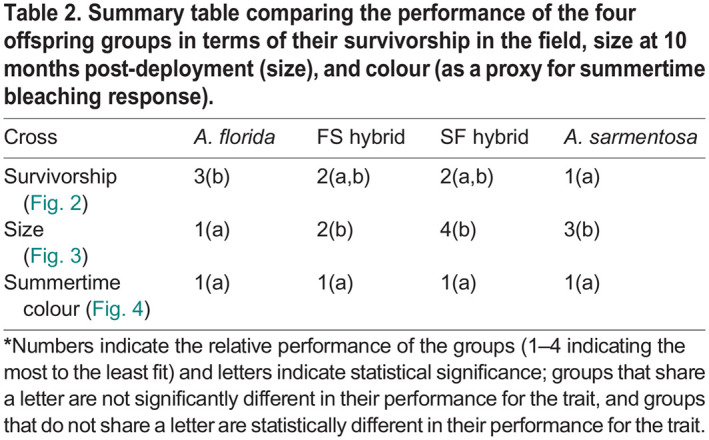
Summary table comparing the performance of the four offspring groups in terms of their survivorship in the field, size at 10 months post-deployment (size), and colour (as a proxy for summertime bleaching response).

### Symbiodiniaceae symbiont communities

**Fig. 5.**
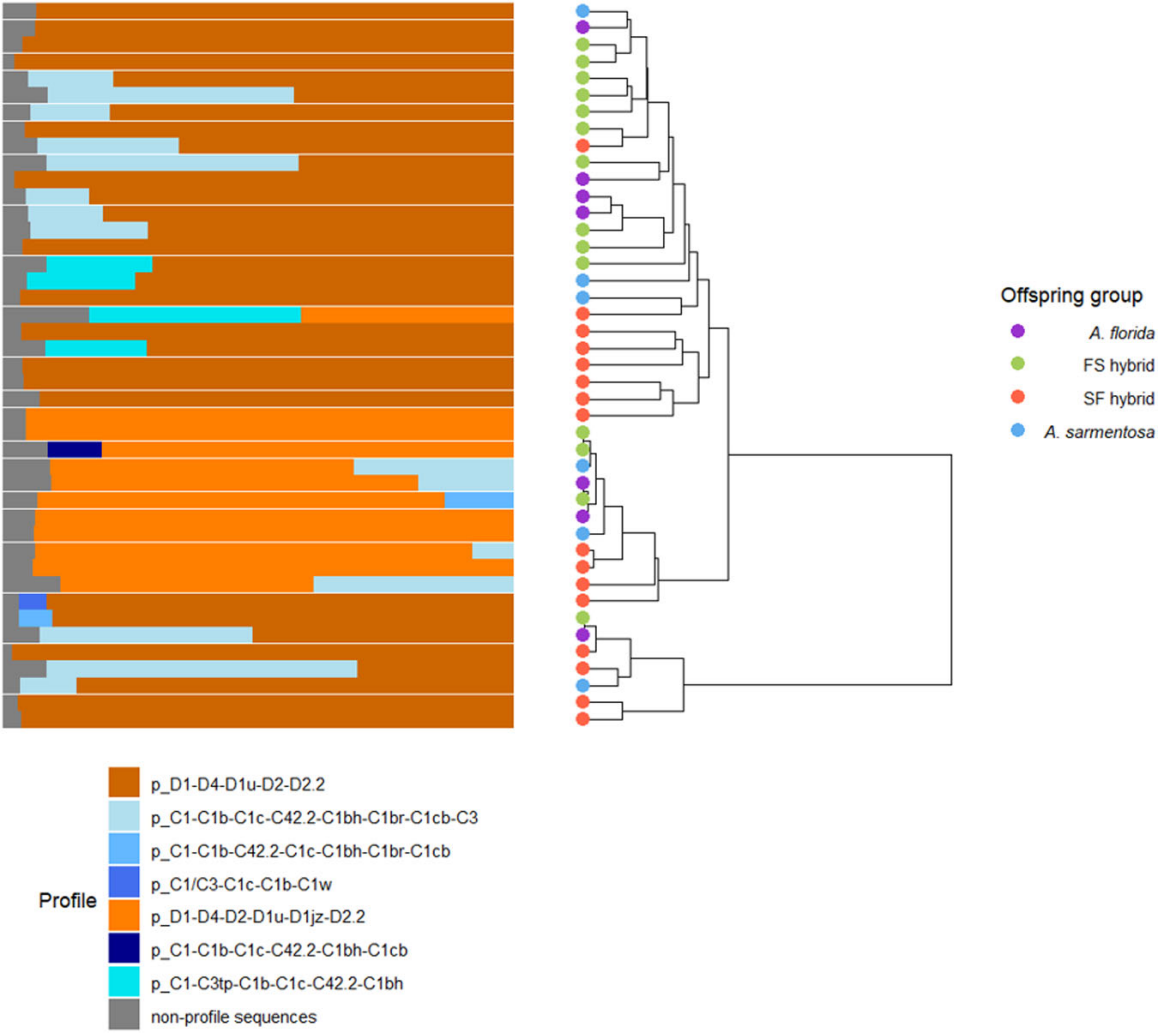
**Symbiodiniaceae community information where each aligned row/tree branch represents data from one sample.** The bar plot shows the relative abundance of Symbiodiniaceae profiles that are putatively characteristic of unique taxa, where each colour represents a profile. A tree visualises the hierarchical clustering of samples according to their unifrac distances, where the tips (representing samples) are coloured by offspring group (*A. florida* purebred – purple, FS hybrid – green, SF hybrid – red, and *A. sarmentosa* purebred – blue).

Metabarcoding using nuclear rDNA internal transcribed spacer 2 (ITS2) sequence data was conducted on the offspring to compare the Symbiodiniaceae communities of the offspring groups. The samples had read (sequencing) depths between 1731–6953 reads. A total of 181887 reads were obtained for the 43 samples analysed for their Symbiodiniaceae communities, averaging 6118, 3989, 2326, and 4378 reads per sample from the *A. florida*, FS hybrid, SF hybrid, and *A. sarmentosa* offspring groups, respectively. At 10 months post-deployment, the surviving coral population had ITS2 profiles that were characteristic of the Symbiodiniaceae general *Cladocopium* and *Durusdinium*. Some samples contained sequences exclusively from the genus *Durusdinium*, while others had mixed *Durusdinium-Cladocopium* ITS2 sequence and DIV profile compositions (Fig. 5). Two *Durusdinium* profiles were detected, distinguished by minor DIV sequence D1jz. Five *Cladocopium* profiles were detected: four characterised by the C1 majority ITS2 sequence, and one with codominant C1/C3 ITS2 sequences. There was no clustering of the offspring groups by Symbiodiniaceae community (R^2^=0.081; *P*=0.318; Fig. 5).

## DISCUSSION

Interspecific hybridisation has been considered as a method to increase genetic diversity and climate resilience in corals (Chan et al., 2019a; van Oppen et al., 2015). However, concerns have been raised that hybridisation for reef restoration could be detrimental to native populations by either reducing population fitness through outbreeding depression or by introducing competition (Aitken and Whitlock, 2013; National Academies of Sciences, 2019). The findings of this study show that F1 hybrids of *A. florida* and *A. sarmentosa* displayed no outbreeding depression or overdominance relative to both of their purebred counterparts in a natural environment.

### Symbiodiniaceae communities

The hybrid and purebred corals showed no difference in symbiont ITS2 type profiles, indicating that observed differences in the performance of the offspring groups were not driven by their Symbiodiniaceae communities. Strain-level variation (variation within Symbiodiniaceae radiations) among the Symbiodiniaceae communities of the offspring groups was not assessed here and can result in differential performance of symbionts, although this typically manifests at larger spatial scales (Howells et al., 2012). The corals were populated by *Durusdinium* or *Durusdinium* and *Cladocopium* after 10 months in the ocean, when the corals were 20 months old. This is in keeping with published results from research on the reefs at Yunbenun that 52 species of cnidarian hosts harbour *Durusdinium* and *Cladocopium* (Abrego et al., 2009) and that, more specifically, multiple Acroporidae species are populated by *Durusdinium* and *Cladocopium* of the C1 radiation (Ulstrup and van Oppen, 2003; van Oppen et al., 2001; Little et al., 2004). Furthermore, Chan et al. (2019b) found that corals of the same purebred and hybrid lineages that were tested here and growing under ambient (27°C, 415 ppm) and elevated (28°C, 685 ppm) temperature and *p*CO_2_ conditions in aquaria were also populated by *Cladocopium* and *Durusdinium*. The dominant *Cladocopium* ITS2 sequences were C3k, Cspc and C33 in the aquarium-reared corals in Chan et al. (2019b) but various sequences from the C1 radiation were typical of *Cladocopium* in the field-reared corals studied here. The corals in this study were derived from spawned gametes of corals from Davies Reef and exposed to symbionts isolated from the tissue of adult colonies from Yunbenun, whilst the corals in Chan et al. (2019b) were parented by corals from Trunk Reef (∼100 km to the north-west of Davies Reef) and exposed to the symbionts isolated from tissue of their parents. In Chan et al. (2019b), 2-year-old *A. florida*, *A. sarmentosa*, FS hybrid, and SF hybrid recruits had established parent-like communities dominated by Symbiodiniaceae with sequence variants C3k, Cspc, and C33. Although the Symbiodiniaceae communities of the donor colonies from Yunbenun in this study were not analysed, previous work indicates that *Cladocopium* that carry the C1 ITS2 sequence are prevalent in acroporid corals in this region (van Oppen et al., 2001; Abrego et al., 2009). The recruits in Chan et al. (2019b) and in this study were therefore likely to have been exposed to different Symbiodiniaceae upon settlement and throughout their lifespans.

### Trade-offs between traits

Comparing the hybrid and purebred offspring groups revealed trade-offs between traits. Here, *A. florida* purebreds were larger at 10 months post deployment (although low sample sizes could have impacted this result) but had the lowest survivorship compared with the other offspring groups. A trade-off between coral growth and survivorship has previously been observed in *Acropora tenuis* deployed in the ocean (Quigley et al., 2021), while a positive relationship between growth and survivorship was detected in *Acropora millepora* exposed to disease, temperature, and acidity stressors in a laboratory environment (Wright et al., 2019); these conflicting results may be due to genetic linkage groups differing between *Acropora* species or the experimental methods and/or the environment–trait interactions differing between these studies. Additional trait trade-offs have been observed in corals: between bleaching resilience and disease resilience (Muller et al., 2018), growth rate and tissue loss following a temperature stress event (Ladd et al., 2017), and growth at ambient temperatures and thermal bleaching tolerance (Little et al., 2004; Jones and Berkelmans, 2011; Cunning et al., 2015). In contrast, Muller et al. (2021) did not detect trade-offs amongst 12 host, symbiont, and holobiont traits in *A. cervicornis* grown under control and elevated temperatures and *p*CO_2_ levels. Trait trade-offs are complex, and currently there is limited understanding of their impact on the adaptive capacity of corals. Further research that compares the effects of environment, experimental methodology, and genetic background on the relative performance of coral treatment groups across traits would enhance our ability to wholistically assess and develop optimum strategies for reef restoration.

### Performance trade-offs in different environments and experiments

Comparing the performance of *A. florida*, FS hybrid, SF hybrid, and *A. sarmentosa* offspring groups in this field experiment with those from a lab-based study revealed environmental trade-offs. Under controlled ambient laboratory conditions (27°C, 415 ppm) in Chan et al. (2018), FS and SF hybrids showed overdominance relative to their purebred counterparts in that they had greater survivorship up until 28-weeks post-settlement. Under elevated temperatures and *p*CO_2_ levels, only the FS hybrids survived better than the *A. sarmentosa* purebreds over the same time (Chan et al., 2018). In contrast to these results from Chan et al. (2018), in the present field-based study, neither hybrid group survived better than the *A. sarmentosa* purebreds and both hybrids showed a trend, although not statistically significant, of better long-term survivorship than the *A. florida* purebreds.

The relative size of the four offspring groups differed between laboratory and field environments. Under ambient conditions in the laboratory, the hybrids grew larger than *A. sarmentosa* purebreds and to the same size as *A. florida* purebreds up until 28-weeks post-settlement (Chan et al., 2018). Under elevated temperatures and *p*CO_2_ conditions in the laboratory, there was no difference in the size of the offspring groups up until 28-weeks post-settlement (Chan et al., 2018). After 1 year in the same experiment, by which time the corals were all held under ambient conditions, there were no surviving *A. florida* recruits (in keeping with the relatively low survivorship of the *A. florida* offspring group seen here) and only two surviving SF hybrids, and the surviving FS hybrids were larger than (presenting overdominance relative to) the *A. sarmentosa* purebreds (Chan et al., 2018). After 10 months in the field, the hybrids in the present study were the same size as the *A. sarmentosa* purebreds and smaller than the *A. florida* purebreds, demonstrating inheritance of the *A. sarmentosa* growth rate. Again, however, it must be noted that the size of relatively few recruits was measured at 10 months post-deployment in the field and this may have affected the validity of this result.

Environmental trade-offs can occur due to antagonistic pleiotropy, when the same genes can increase the fitness of an organism in one environment but decrease its fitness in another, and conditional neutrality, where an allele has a positive fitness effect in one environment and a neutral fitness effect in another environment (Anderson et al., 2013). Environmental trade-offs have been demonstrated in transplantation studies of corals where their performance in one environment differs to their performance in a different environment (Mieog et al., 2009; Kenkel et al., 2015; Quigley et al., 2021). Environmental trade-offs may be a driver in observed patterns of hybrid and purebred relative abundance, survivorship, and growth differing amongst reef zones (Willis et al., 2006; Fogarty, 2012). The difference in the relative performance of the hybrid and purebred corals studied here in the field and in Chan et al. (2018) in an aquarium is indicative that laboratory-based studies may not adequately predict field success and that hybrid performance is likely to vary between environments (National Academies of Sciences, 2019; O'Donnell et al., 2018). This highlights the importance of field-testing restoration approaches to better predict the benefits and risks of interventions.

Furthermore, the corals in this field trial and those in the laboratory experiment by Chan et al. (2018) were studied over different periods of their lives, had parents from different reefs on the GBR, and harboured different symbiont communities; these differences could have affected the relative performance of the offspring groups. Variation in cross-fertilisation compatibility and parental genotypes within the intraspecific and intraspecific crosses conducted here and elsewhere may have also resulted in replicated offspring groups that differed in their genetic diversity and thereby performance. It must further be noted that *A. sarmentosa* and *A. florida* have overlapping spawning times and distributions (demonstrated here and in Chan et al., 2018 and Baird et al., 2021), are closely related (Cowman et al., 2020), and are highly cross-fertile (demonstrated here and in Chan et al., 2018) such that hybridisation may naturally occur between these species. If cryptic hybridisation occurs between these species, then first or later generation hybrids may have been included in the broodstock in this experiment and this would affect the hybrid versus purebred comparisons that have been drawn. However, morphologically distinct *A. florida* and *A. sarmentosa* populations were collected and bred, and the morphology of other acroporid hybrids has been demonstrated to be intermediate and not cryptic compared to their purebred parental species (Fogarty, 2012; Isomura et al., 2013).

It should be noted that the corals in this field experiment were grown in aquaria for the first 10 months of their lives prior to deployment and therefore experienced a significant shift in environment throughout their lifespan and different selective pressures pre- and post-deployment. The offspring groups displayed equal pre-deployment survivorship that was variable among tanks potentially due to differences in the tank environments (since the tanks likely contained different biological communities). The aquarium environment/s may have selected for traits in the coral populations studied here, which may have subsequently impacted their performance in the field (López-Nandam et al., 2022).

### Implications for interspecific hybridisation as a restoration tool

F1 hybrids that have higher fitness than purebreds under stressful conditions could constitute resilient coral stock that might boost the resilience of actively restored reefs. Hybrids have performed better than one or both of their purebred parental species based on their size and survivorship under ambient conditions, and size under elevated temperature and *p*CO_2_ conditions in the laboratory (Chan et al., 2018), survivorship and growth on some reef zones (Fogarty, 2012), growth in coral nurseries (VanWynen et al., 2021), and resistance to disease, predation, and parasitism (Fogarty, 2012). Conversely, hybrids have performed worse than one of their parental species in terms of their size (albeit derived from low sample sizes) in this study, and growth and survivorship on some reef zones (Fogarty, 2012; Willis et al., 2006). These results indicate that hybrid corals tested to date do not have enhanced fitness across all traits or contexts and thus their potential to boost the resilience of reefs has limits. However, in the Caribbean, the coral hybrid *A. prolifera* exists outside the spatial distribution range of, and is increasing in relative abundance at, some sites compared to its purebred parental species, which have experienced serious declines over recent decades (Fogarty, 2012; Willis et al., 2006). This indicates that some coral hybrids can be beneficial to restoring degraded reefs. The species studied here, *A. florida* and *A. sarmentosa*, have different morphological and ecological traits such that hybrids between them might occupy unique niches and fulfil unique functional roles within reef ecosystems (Madin et al., 2023; McWilliam et al., 2018). F1 hybrids also constitute a potential tool to boost the genetic diversity of coral stock and thereby the adaptive potential of actively restored reefs. Future research that compares the trait space occupancy and genomes of these and other hybrids would provide valuable insights into the diversity hybrids can contribute to reef ecosystems. Reduced fitness of F1 hybrids relative to both purebred counterparts has not yet been observed in corals, indicating that the risk of outbreeding depression in F1 coral hybrids is low (Willis et al., 2006; Chan et al., 2018; Fogarty, 2012). Coral hybrids have also not performed consistently and in an overdominant manner across environments and fitness traits, indicating the risk of hybrids rapidly, widely, and problematically outcompeting their purebred parental species is low (Willis et al., 2006; Chan et al., 2018; Fogarty, 2012). The high fertilisation success of some hybrid crosses and lack of evidence of outbreeding depression and overdominance in F1 hybrids indicates that interspecific hybridisation could be a low-risk approach to generating genetic diversity and adaptive potential in reef-building corals (Willis et al., 2006; Chan et al., 2018; Fogarty, 2012). Although, the inconsistency of hybrid performance across environments that has been observed to date indicates that coral hybrids must be further deployed directly onto multiple reef environments and studied for the value of hybrid stock to reef restoration initiatives to be comprehensively assessed. In cases where the performance of a coral hybrid is greater than that of one of its purebred parental species, those hybrids further constitute a way to conserve the genetic information of the poorer-performing parental species through climate change-induced stressors. However, restoration programs considering deploying hybrid stock to achieve conservation goals must balance the risks of losing genetic uniqueness among species against the benefits of enhancing the potential for species to adapt and persist in future climates (Allendorf et al., 2001).

## MATERIALS AND METHODS

### Species

*A. florida* and *A. sarmentosa* were selected for this study because acroporids are widespread and common reef-building species (Wallace, 1999). Furthermore, these two species are morphologically distinct, can spawn at the same time (Baird et al., 2021), have highly cross-fertile gametes (Chan et al., 2018), and the performance of their interspecific hybrids has been tested under a variety of conditions in the laboratory (Chan et al., 2018, 2019c). Therefore, the interspecific hybrids of *A. florida* and *A. sarmentosa* are model offspring groups for investigating coral hybrids because they can be consistently produced, and analysing their field performance will add to a growing body of knowledge that will enable holistic assessment of their value as coral stock for restoration initiatives.

### Coral stock generation and captive rearing

Gravid colonies of *A. florida* and *A. sarmentosa* colonies were collected (GBRMPA Permit G12-35236.1) on the 14/11/2019 from Davies Reef (18.828 S, 147.643 E), during the GBR mass spawning event of November 2019 (12/11/2019 full moon). The colonies were temporarily housed in temperature-controlled outdoor aquaria under natural light at the National Sea Simulator (SeaSim) at the Australian Institute of Marine Science (AIMS; Townsville, Australia) and monitored for spawning. Colonies were isolated upon showing signs of ‘setting’ and imminent spawning (Harrison et al., 1984).

Following spawning on the night of the 20 November 2019, gamete bundles from each colony were collected and the eggs and sperm separated and isolated using a 100 μm mesh filter. Briefly, the sperm from all colonies from the same *Acropora* species were combined to create a mixed sperm solution with equal density of sperm from each donor colony; thus, two mixed sperm solutions were created – one for *A. florida* and one for *A. sarmentosa*. The mixed sperm solution was added to the eggs of each conspecific colony to generate purebreds and to the eggs of each colony of the other species to generate hybrids at an approximate density of 1×10^6^ sperm mL^−1^ (Table 1). Note that using this method of crossing and in the purebred crosses, the eggs of a colony were exposed to the sperm of the same colony such that self-fertilisation was possible.

At 2.5 h post-fertilisation, three samples of 100 eggs from each cross between the eggs of one dam and a mixed sperm solution were checked for fertilisation success based on the proportion of multi-cell embryos and unfertilised (undivided) eggs, and fertilisation success was graphed on a boxplot and compared among offspring groups using R (R Core Team, 2022). A generalised linear mixed effects model (GLMM) was built using the lme4 package (Bates et al., 2014) to test the effect of offspring group on the number of embryos undergoing cell division at 2.5 h. Dam was included in the model as a random effect and a Poisson link function was applied. A *post-hoc* Tukey's test was conducted to compare fertilisation success amongst the offspring groups using the multcomp package (Hothorn et al., 2008).

Post-fertilisation, the embryos were combined according to the four offspring groups (Table 1) and maintained in larval rearing tanks for approximately 1 week until they became competent to settle. Once competent, the planula larvae were introduced to aquaria with 100×100 mm terracotta tiles that had been biologically conditioned in coral rearing tanks for 6 weeks to enhance larval settlement. The conditioned tiles were placed in 50 L or 200 L tanks where they were raised slightly off the bottom or leant against the walls. Larvae were added to the tanks at an approximate density of 200 larvae per tile. Larvae from the different offspring groups were settled in separate tanks such that, post-settlement, each tile housed corals of just one offspring group; settled coral juveniles are hereafter referred to as recruits. In the settlement tanks, the larvae from the different offspring groups were exposed to equal densities of photosymbionts (Symbiodiniaceae; 2×10^6^ cells mL^−1^) that had been isolated from fragments of adult *A. tenuis* colonies from the outplant location, the reefs fringing Yunbenun (Magnetic Island), using the approach outlined in Chan et al. (2018). The larvae and recruits were also exposed to the Symbiodiniaceae that were present on the conditioned tiles and in the water of their rearing tanks.

The coral recruits were reared amongst three 500 L tanks in outdoor systems at AIMS under natural light for ten months before they were deployed. Over a period of 47 days, beginning approximately seven weeks post-settlement, the recruits growing on a subset of the terracotta tiles were censused under a dissecting microscope: 27, 69, 48, and 58 tiles of *A. florida*, FS hybrids, SF hybrids and *A. sarmentosa* recruits were censused, respectively. The live recruits at this initial time point were compared to those censused on the same tiles 10-months post-settlement and immediately prior to their deployment to obtain estimates of early life stage survivorship of the recruits in the SeaSim.

The survivorship of each coral over the period they were reared in the SeaSim was scored as a binary response – dead or alive. To test the effect of offspring group on survivorship, a GLMM was constructed using the lme4 package in R using a Bernoulli link function. A model was built that tested the fixed effect of offspring group on survivorship while accounting for the random effects of tile nested within tank and date of the initial census. The emmeans function of the emmeans package (Lenth, 2022) was used to compare early life survivorship among the offspring groups and a Bonferroni correction was applied to account for multiple comparisons.

### Coral deployment

Deployment of recruits was planned to occur early in 2020 however COVID pandemic restrictions on travel and fieldwork delayed deployments. In September 2020, tiles were assessed and those containing live recruits were identified and randomly grouped into sets of 12. The tiles, with holes in their centres, were threaded onto stainless steel rods in sets of six, separated by 4 cm PVC spacers. Each pair of rods was loaded into a PVC cassette for ease of handling (Fig. S1). The cassettes were transported to Geoffrey Bay, Yunbenun (-19.1565 S, 146.8642 E), on the 20/09/2020 (when the corals were 10 months of age), attached to four fibreglass reinforced plastic frames (220 cm long×120 cm), and raised 62.5 cm off the substrate (GBRMPA Permit G19/42928.1; Fig. S1). The frames were deployed at ∼5–10 meters depth (tide dependent), where they remained submerged during low tides and where acroporid corals naturally occur. Geoffrey Bay was chosen as the deployment site because of its accessibility from AIMS and, as an inshore location, it experiences higher temperatures than offshore locations such that the deployed corals were more likely to be tested on their performance during thermal stress events. The frames were secured into the sandy substrate using star pickets. The corals were randomised across the frames such that each frame contained an approximately equal subset (numbers of tiles) from each offspring group.

### Censusing and statistical analyses

The corals were censused over a maximum of 7 days and the performance of the offspring groups was compared at 2 weeks prior to deployment (September 2020) and at approximately 2 (November 2020), 4 (January 2021), 6 (March 2021), and 10 months (July 2021) post-deployment. Throughout the course of the experiment, corals that grew into physical contact with one another were excluded from further analyses because they could not be considered independent replicates. Note that one of the four frames was damaged and turned over (possibly by rough weather or anchor hook-up) between 4–6 months post deployment and another between 6–10 months post-deployment; the corals growing on these frames were excluded from further analyses.

Statistical analyses and graphs were conducted and produced using R. Unless otherwise stated, graphics were generated using the ggplot2 package (Wickham, 2016).

#### Survivorship

The survivorship of each coral was scored at each time point as a binary response: dead or alive. To test the effect of offspring group, time, and an interaction between offspring group and time on survivorship, BGLMMs were constructed using the brms package in R using a Bernoulli link function (Bürkner, 2017). A model was built that accounted for the repeated measures design and included the fixed effects of time (factor), offspring group, and an interaction between the two; models that also included the random effects of tile and/or frame (tile was nested within frame when both were included) were also constructed. Four chains were run for each model for 5000 iterations with a 2000 iteration warm-up. The models were compared to the simpler model using the LOO package (Vehtari et al., 2017) and the simplest model that was not outperformed by any more complex model was considered the best performing. The emmeans function was used to compare the estimated marginal mean survivorship among the offspring groups at each time point based on the best performing BGLMM and a Bonferroni correction was applied to account for multiple comparisons. Kaplan–Meier survival probabilities at each time point were further calculated for each offspring group and graphed using the survival (Therneau, 2015) and survminer (Kassambara et al., 2017) packages. The survivorship curves of the different offspring groups were compared using log-rank tests and the *P*-values were adjusted using a Benjamini–Hochberg method to control for the false discovery rate (Benjamini and Hochberg, 1995).

#### Size

The tiles were imaged using a high-resolution camera (Nikon D810) prior to deployment and at 2- and 4-months post-deployment. The imaging stage included a scale bar and the D-side of the Coral Watch Coral Health Chart (Siebeck et al., 2008) for size and colour measurements (below), respectively. The images were analysed using ImageJ software (Schneider et al., 2012). The images were scaled using the scale bar to ensure accurate size measurements. Corals were circled using the freehand selection tool in ImageJ and 2D recruit surface area (mm^2^) was measured. By 6 months post-deployment, many corals had complex 3D structures and so 2D surface area was no longer an accurate indication of size. At 10 months post deployment, the surviving corals were 3D-imaged and modelled for surface area calculations using an approach based on the methodology outlined in Ferrari et al. (2017). Briefly, the corals were photographed using the Olympus Tough TG-6 with coded markers. The number of photos taken of a coral was dependent on its size and complexity. Models were constructed from the images using Agisoft Metashape 1.6.4 (Agisoft LLC, 2021). The standard photogrammetric workflow – ‘Align Photos’, ‘Optimize Photo Alignment’, ‘Build Dense Cloud’, ‘Build Mesh’, and ‘Build Texture’ (in order) – was followed using the default settings. The images were scaled using six markers which formed three scale bars of known length. The free-form tool was used to ‘select’ the coral and its surface area was then calculated.

Linear mixed effects models (LMM) were used to compare the size of the corals among offspring groups over time. Models were constructed using the nlme package (Pinheiro et al., 2022) to test the effect of offspring group and time (factor), and the interaction between the two, on recruit surface area. The repeated measures design and the increase in coral size variance over time were accounted for; an increase in coral size variance over time is expected due to some recruits growing more than others and because of partial colony mortality events. Various models also accounted for either or both the non-independence of corals growing on the same tile and frame. Likelihood ratio tests were used to compare the performance of the models and the simplest model that was not outperformed by any more complex model was considered the best performing. The emmeans function was used to compare the estimated marginal mean sizes of the recruits of each offspring group at each time point and between consecutive timepoints for the same offspring group and a Bonferroni correction was applied to account for multiple (*N*=36) comparisons.

#### Site temperature tracking and bleaching response

The temperature at 5 m depth in Geoffrey Bay was recorded at 5-min intervals by loggers that were deployed as a part of the Australian Institute of Marine Science Temperature Logger Program (Australian Institute of Marine Science, 2017). The site temperature data was retrieved from deployment on the 20 September 2020 until the 6 June 2021. The mean, minimum, and maximum daily temperatures throughout the course of the deployment were calculated and plotted. The water temperatures in Geoffrey Bay over this period were compared to the historical temperatures of Davies Reef – where the parental colonies were collected from – to gauge the level of temperature stress experienced by the deployed recruits. The 40-year average maximum 6 m depth water temperature of the hottest month, February, was calculated for Davies Reef (Australian Institute of Marine Science, 2017). The temperatures in Geoffrey Bay were compared to this Davies Reef summer maximum and summed over time to calculate the number of degree heating weeks (DHWs) cumulatively experienced by the recruits using the approach outlined by the NOAA Coral Reef Watch Bleaching Alert System (Heron et al., 2014). The cumulative DHWs experienced by the recruits were graphed over time.

The 2D high resolution images that were taken prior to deployment and at 2 and 4 months post-deployment were sampled for greyscale measurements. At 6 and 10 months post-deployment, the corals were imaged using the TG-6 from the viewpoint that captured the largest surface area of the coral with the D-side of the Coral Watch Coral Health Chart. Each image was converted into 8-bit and calibrated using the colour chart. The corals were circled using the freehand tool and the calibrated colour score was measured across the surface area of each of the recruits as a proxy for bleaching response. The colour score of the corals was tracked over time and the change in colour score was compared among offspring groups using linear mixed effects modelling. Note that, because two species with different colourations and appearance and their interspecific hybrids were included in this study, relative colour scores were tracked by dividing the colour score of the recruit at each time point by the colour score of the same recruit prior to deployment. Models were built and compared using the same approach as for the analysis of coral growth over time (see above). The emmeans function was used to assess the change in colour within each offspring group between consecutive timepoints and a Bonferroni correction was applied to account for multiple comparisons.

### Symbiodiniaceae symbiont communities

At 10 months post-deployment, tissue was sampled from the surviving corals and placed into absolute ethanol for classification of their Symbiodiniaceae communities based on sequence data of the ITS2 region. Holobiont (including coral and Symbiodiniaceae) DNA was extracted following the protocol outlined in Damjanovic et al. (2019). The ITS2 region was amplified using the SYM_VAR_5.8S2/SYM_VAR_REV primers (Hume et al., 2013, 2015) and optimised protocol outlined in (Hume et al., 2018). The PCR reaction of each sample was performed in triplicate and the triplicates were then pooled. Library preparation was conducted on the samples following Maire et al. (2021) and paired-end (2×300 bp) sequencing was conducted using one MiSeq V3 system (Illumina) at the Walter and Eliza Hall Institute of Medical Research (Melbourne, Australia). The sequences were analysed using the SymPortal analytical framework (Hume et al., 2019). SymPortal handles Symbiodiniaceae intragenomic ITS2 variants by identifying within–sample ITS2 defining intragenomic variants (DIVs) and using them to characterise ITS2 profiles that are putatively representative of Symbiodiniaceae taxa. The raw sequencing data underwent quality control and analyses standard to the SymPortal framework (Hume et al., 2019). SymPortal quality-controlled ITS2 sequences and ITS2 profile abundance data was subsequently analysed in R. The relative abundance of the ITS2 profiles present in each sample were plotted. A pairwise kmer-based similarity matrix amongst the sequences was generated using a *k* size of seven (Fujise et al., 2021) in the kmer package (Wilkinson, 2018), and a hierarchical clustering approach was used to generate a phylogenetically informed dendrogram from the distance matrix using the upgma function from the phangorn package (Schliep, 2011). Weighted unique fraction metric (UniFrac) distances (Lozupone and Knight, 2005) were then calculated amongst samples based on the presence, abundance, and relatedness of the ITS2 sequences present in each sample and the phylogenetic tree. The ggtree package (Yu et al., 2017) was used to create a tree that visualised the hierarchical clustering of samples, in which samples were coloured by offspring group. PERMANOVA was then used to test for the effect of offspring group on UniFrac distance, while accounting for the nesting within frame using the package Vegan (Oksanen et al., 2020).

## Supplementary Material

10.1242/biolopen.060482_sup1 Supplementary information
