# Supplementary material for: Interspecific hybridisation provides a low-risk option for increasing genetic diversity of reef-building corals
Source: Biol Open. 2024 Aug 29;13(9):bio060482. doi: 10.1242/bio.060482 (PMC11381923; doi:10.1242/bio.060482)
Supplement: Supplementary information [file biolopen-13-060482-s1.pdf]

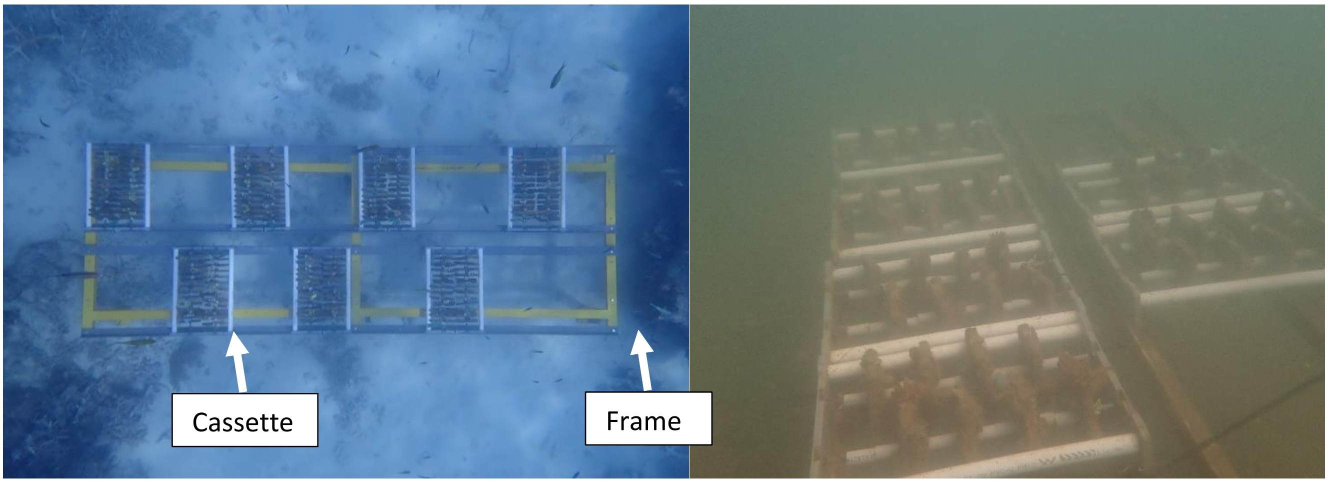

Fig. S1. Images showing the tile deployment design. The left image was taken from an earlier deployment that utilised the same experimental design. Images of coral hybrids at the Yunbenun site were poor quality due to low visibility. The right image shows the tile arrangement on the cassettes at Yunbenun. Tiles containing corals were randomly grouped into sets of 12 and threaded onto stainless steel rods in sets of six, separated by 4 cm PVC spacers. Each pair of rods was loaded into a PVC cassette for ease of handling. The cassettes were attached to four reenforced fibreglass plastic frames that were 220 cm long, 120 cm wide, and raised 62.5 cm off the substrate at 5 – 10 metres depth (tide-dependent).

Table S1. Comparisons of the predictive capacity of Bayesian generalised linear mixed effects models built to test the effect of time (continuous), offspring group, and an interaction between the two on coral survivorship. Random factors are denoted using the “1|” annotation. All models accounted for the repeated measures design (1|CoralID) and some models accounted for random variation amongst tiles (1|TileID) and frames (1|Frame). The Bayesian LOO estimate of the expected log pointwise predictive density (elpd_loo) is shown for each model. The difference in elpd_loo (elpd_diff) and standard error in the difference (se_diff) are shown for each model comparison. The better performing model is listed as model 1 in each pairwise comparison. The model that accounted for the repeated measures design and included the fixed effects of time, offspring group, and an interaction between the two performed better than the models that also included tile or tile and frame as random effects and no differently than the model that included frame as a random effect.

Model 1	Model 1 elpd_loo	Model 2	Model 2 elpd_loo	elpd_diff	se_diff
Survival ~ Offspring Group X Time + (1| CoralID)	-232.6	Survival ~ Offspring Group X Time + (1|TileID/CoralID)	-233.8	-1.1	3.2
Survival ~ Offspring Group X Time + (1| CoralID)	-232.6	Survival ~ Offspring Group X Time + (1|Frame/CoralID)	-234.3	-1.6	2.6
Survival ~ Offspring Group X Time + (1| CoralID)	-232.6	Survival ~ Offspring Group X Time + (1|Frame/TileID/CoralID)	-234.6	-2.0	3.2

Biology Open • Supplementary information
